# Supplementary material for: High-power hybrid biofuel cells using layer-by-layer assembled glucose oxidase-coated metallic cotton fibers
Source: Nat Commun. 2018 Oct 26;9:4479. doi: 10.1038/s41467-018-06994-5 (PMC6203850; doi:10.1038/s41467-018-06994-5)
Supplement: Supplementary file 1 — Supplementary Information [file 41467_2018_6994_MOESM1_ESM.pdf]

Supplementary Information

High-Power Hybrid Biofuel Cells Using Layer-by-Layer Assembled Glucose Oxidase-Coated Metallic Cotton Fibers

Kwon *et al.*

Supplementary Note 1

Electrochemical impedance spectra

The electrochemical impedance spectra of the 5-, 10-, and 20-MCFs shown in **Fig. 2h** displayed similar characteristics, consistent with charge transfer kinetics (a depressed semicircular arc at high frequencies) and diffusive control (a straight tail at low frequencies, called the Warburg impedance). Each impedance component value was obtained from fitting the Randle's equivalent circuit, consisting of R_s, R_{ct}, constant phase element (CPE_{dl}), and W, corresponding to the equivalent series resistance (ESR), the charge transfer resistance, the double-layer capacitance, and the Warburg impedance, respectively (**Supplementary Fig. 6a**). In this case, the CPE_{dl} was used for the double-layer capacitance associated with the interface between the electrolyte and the porous MCF electrodes. With increasing number of bilayers (n) of TOA-Au NP/TREN (n = 5 – 20), the ESR at 1 kHz and the R_{ct} values significantly decreased from 1346 to 30 Ω, and from 303.1 to 3.9 Ω, respectively, indicating that better charge transfer kinetics for the MCFs could be obtained by increasing the loading (or number of bilayers) of the Au NPs, followed by an increase in both the electrical conductivity and the effective surface area. Additionally, the CPE_{dl} value (903.3 μF) for 20-MCF was higher than those of other MCFs (48.1 μF for 5-MCF and 546.2 μF for 10-MCF). This also reflects the increase in the active surface area with the high mass loading of the Au NPs. The frequency-dependent electrochemical behavior of MCFs can further be characterized by the knee frequency, defined as a crossover point from the charge transfer reaction to the diffusion-controlled region. These knee frequencies were obtained from admittance plots (**Supplementary Fig. 6b**). As a result, the knee frequency of the 20-MCF was higher than those of the 5-, and 10-MCFs, implying more facile mass transport during electrochemical operations[1].

Supplementary Fig. 21 shows Warburg impedance coefficient plots of $(GOx/PEI)_{30}$ and $(GOx/TREN)_{30}$ on 20-MCF. The slope of each curve represents the value of the Warburg impedance coefficient (σ_w) that is obtained by plotting the impedance spectra in the low frequency region using Equation:

$$Z' = R_s + R_{ct} + \sigma_w \omega^{-0.5},$$

where ω is the angular frequency.

Additionally, the diffusion coefficient (D) can be calculated using Equation:

$$D = 0.5(RT/AF^2\sigma_w C)^2,$$

where R, T, A, F, and C indicate the gas constant, absolute temperature (K), the electrode area (cm^2), Faraday's constant ($C\ mol^{-1}$), and the molar concentration of ions in the electrolyte ($mol\ l^{-1}$), respectively. Therefore, a decrease in the Warburg impedance coefficient means an increase in ion diffusion through the electrode. As a result, the $(GOx/TREN)_{30}$ multilayers on the 20-MCF exhibited a smaller σ_w value (~37.9 $\Omega\ s^{-0.5}$) than the $(GOx/PEI)_{30}$-based electrode (~$2.929 \times 10^2\ \Omega\ s^{-0.5}$), implying a more facile ion diffusion process. More specifically, the D values of the $(GOx/TREN)_{30}$- and $(GOx/PEI)_{30}$-based electrodes were 3.0×10^{-4} and 5.3×10^{-6} $cm^2\ s^{-1}$, respectively[2].

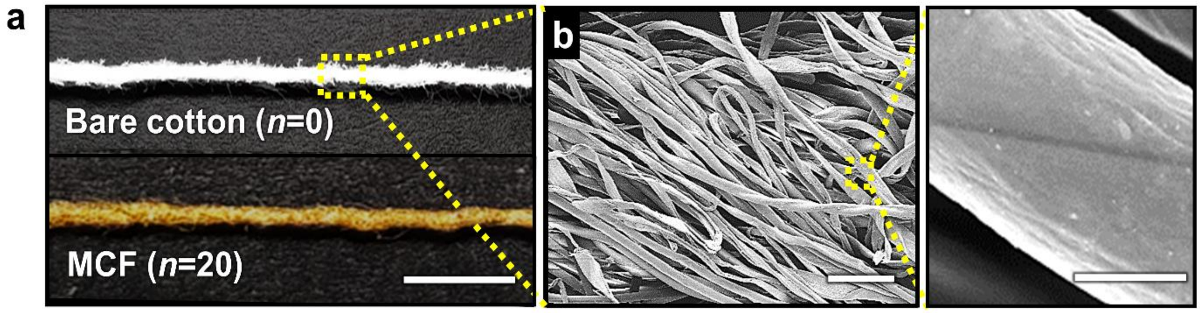

Supplementary Figure 1. Surface morphology of the bare cotton fibers. a, Photographic (scale bar, 1 mm) and **b**, Field-emission scanning electron microscopy (FE-SEM) images of bare cotton fibers (scale bar, 50 μm (left) and 5 μm (right)).

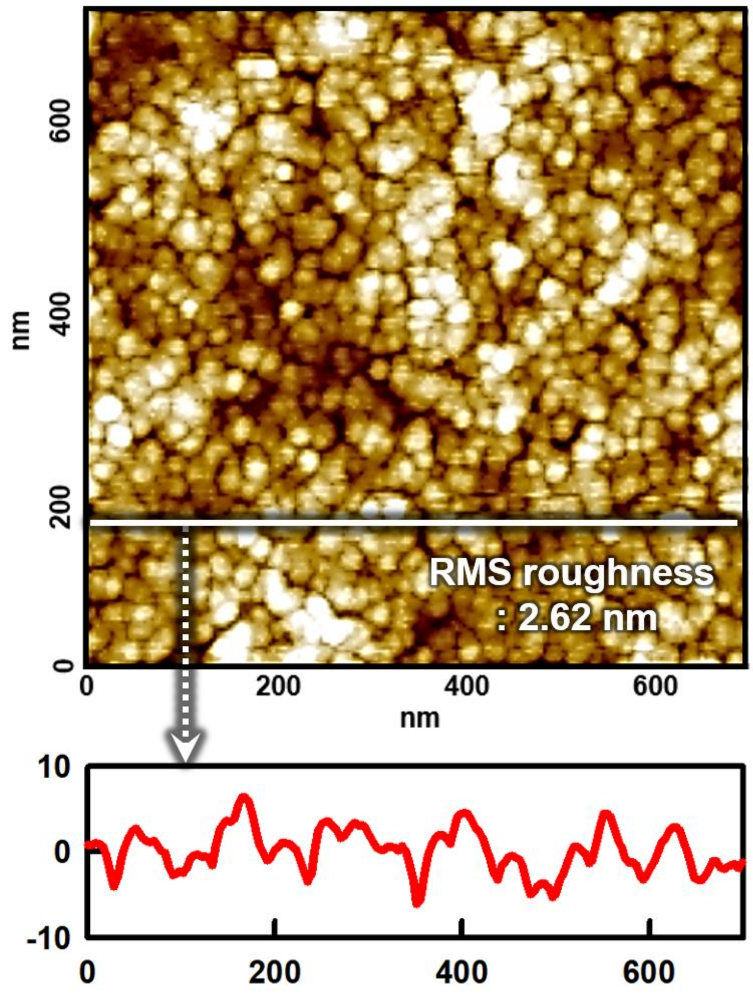

Supplementary Figure 2. AFM image and surface roughness profile of (TOA-Au NP/TREN)$_{20}$ multilayer-coated Si wafer.

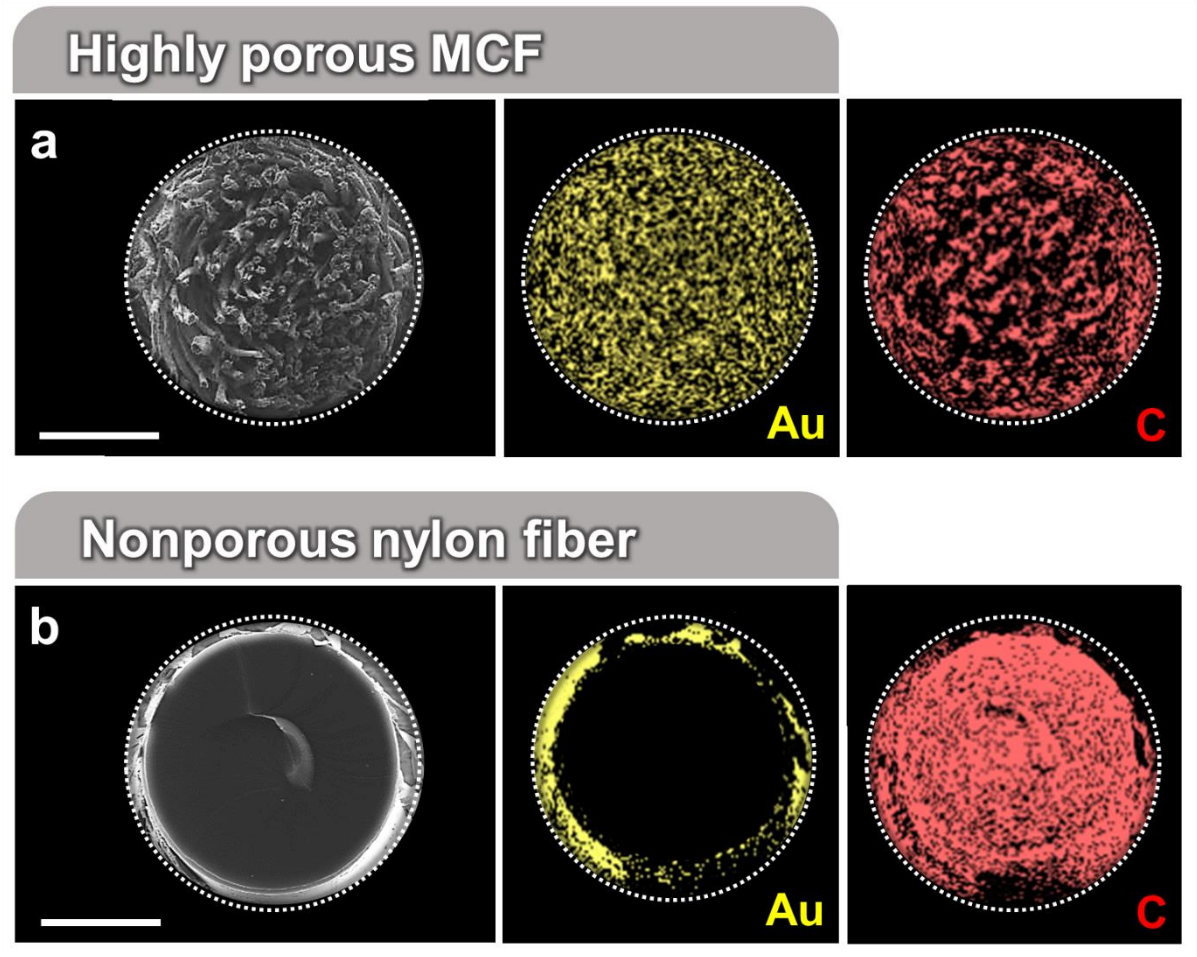

Supplementary Figure 3. Atomic analysis using energy-dispersive X-ray spectroscopy mapping images. Cross-sectional field emission-scanning electron microscopy (FE-SEM) and energy-dispersive X-ray spectroscopy (EDS) images of (TOA-Au NP/TREN)$_{20}$-coated cotton fibers **a**, with a highly porous structure and nylon fibers **b**, with a nonporous structure. As confirmed by the EDS images, the TOA-Au NPs uniformly and deeply infiltrated into the inner center as well as the outer surface of the porous cotton fibers. In contrast, the TOA-Au NPs were deposited only on the outer surface of the nonporous nylon fibers.

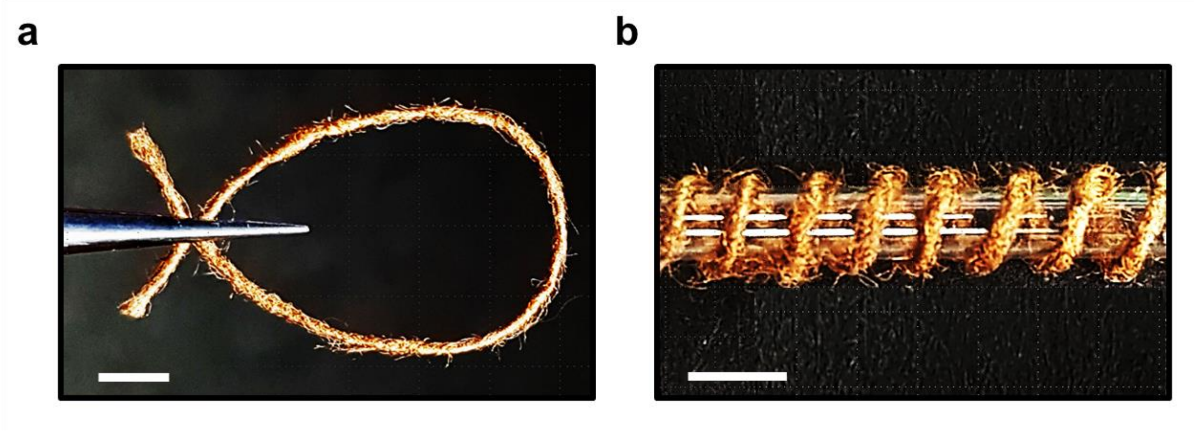

Supplementary Figure 4. Flexibility of 20-MCF. a, Optical image showing a bent MCF electrode, which is held by tweezers. **b**, Optical image of a flexible MCF-BFC electrode in which the 20-MCF is helically wrapped around a 1.5-mm diameter glass rod. All scale bars in FE-SEM images indicate 1 mm.

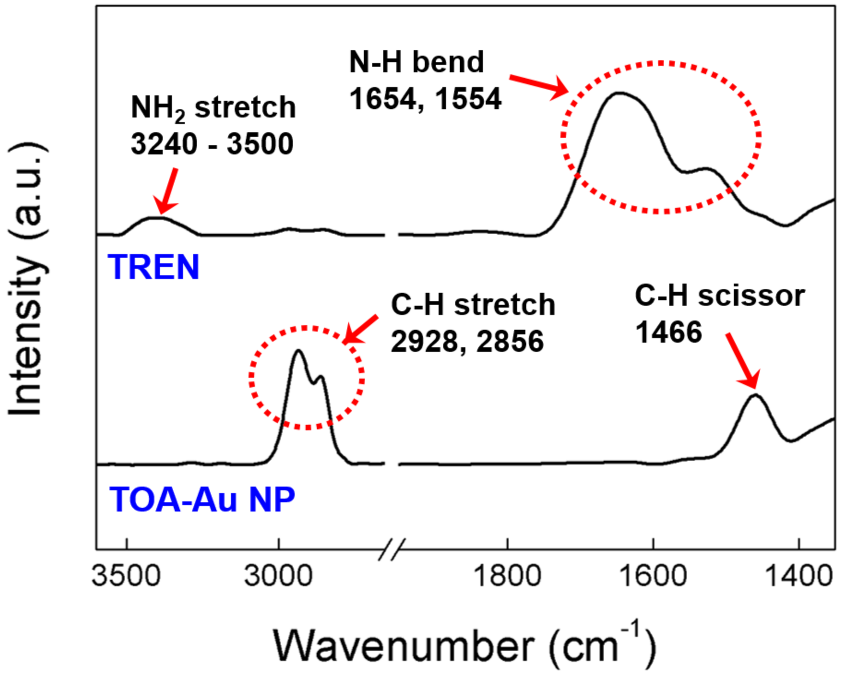

Supplementary Figure 5. Fourier transform infrared spectra characterization. Fourier transform infrared (FTIR) spectra of TREN and TOA-Au NPs. In the case of TOA-Au NPs, FTIR absorbance peaks at 2928 and 2856 cm^{-1} arise due to the C—H stretching of the TOA ligands with long alkyl chains, which were primarily traced to investigate the ligand replacement reaction between the TOA ligands bound to the surface of Au NPs and the NH_2 groups of TREN during LbL assembly.

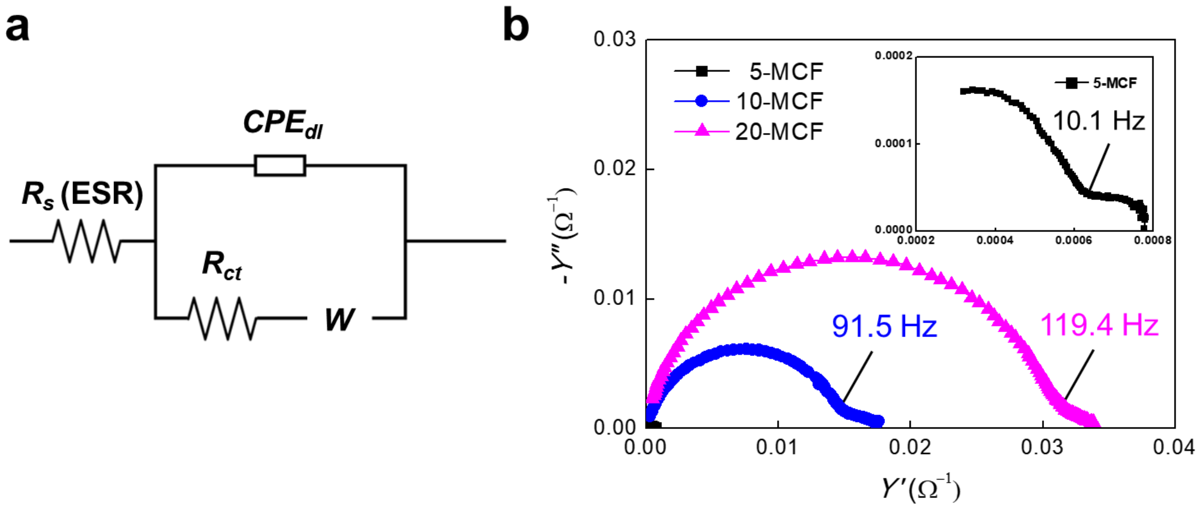

Supplementary Figure 6. Admittance plot for the charge transfer rate related to the diffusion distance with knee frequency. a, Representative equivalent circuit from the Nyquist plots in Fig. 2h. **b**, Admittance plots of MCFs with different numbers of bilayers (*n*). Each frequency values reflects the knee frequencies. Inset: enlarged view of the plot for 5-MCF.

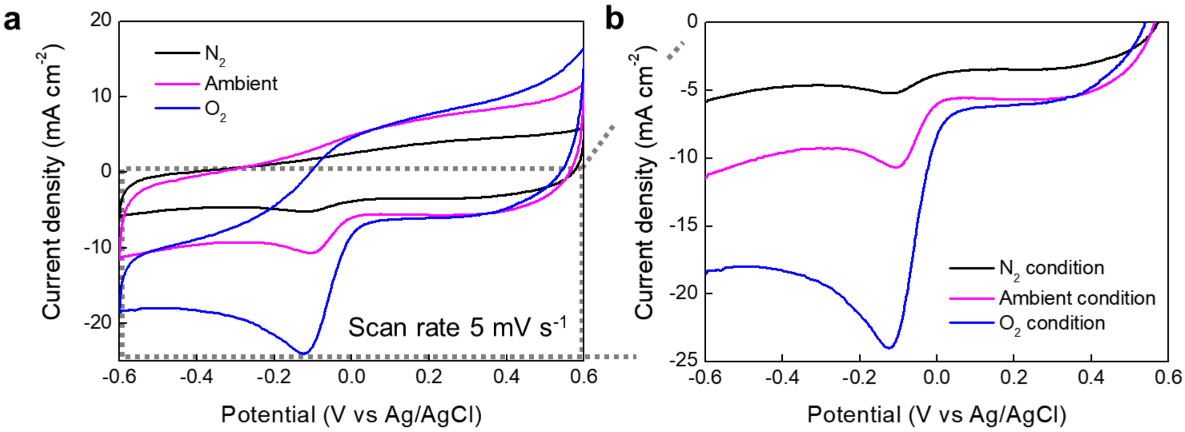

c

Buffer condition	Measured current density level at -0.6 V (mA cm^{-2})	Normalized current density level at -0.6 V (mA cm^{-2})
N_2	-7.2	0
Ambient	-17.1	-10.0
O_2	-27.9	-20.7

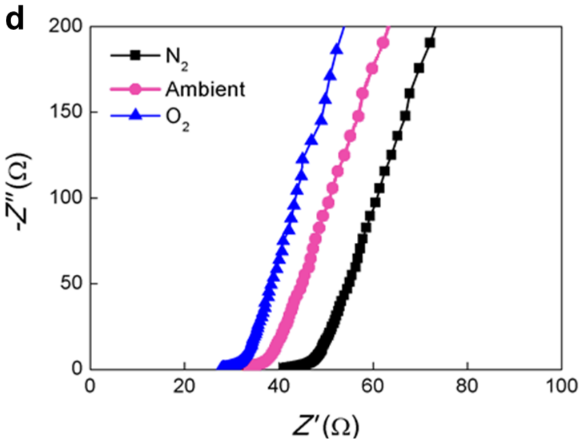

Supplementary Figure 7. Electrochemical performance of 20-bilayered metallic cotton fiber. a, Cyclic voltammograms, **b**, cathodic performance curves, and **c**, normalized areal cathodic current density levels in ambient condition and O_2 conditions. The normalized current density levels (−10.0 mA cm^{-2} under ambient condition, and −20.7 mA cm^{-2} under O_2 condition) were obtained by subtracting the areal cathodic current density level measured at a specific potential of −0.6 V in oxygen-free condition (−7.1 mA cm^{-2}) from the areal cathodic current density level (−17.1 mA cm^{-2} and −27.9 mA cm^{-2}, respectively) measured at the same potential in ambient or oxygen-rich condition. **d**, Nyquist plots of 20-MCF in PBS buffer solution under N_2, ambient, and O_2 conditions. The ESR was 43, 33, and 29 Ω for N_2, ambient, and O_2 conditions, respectively.

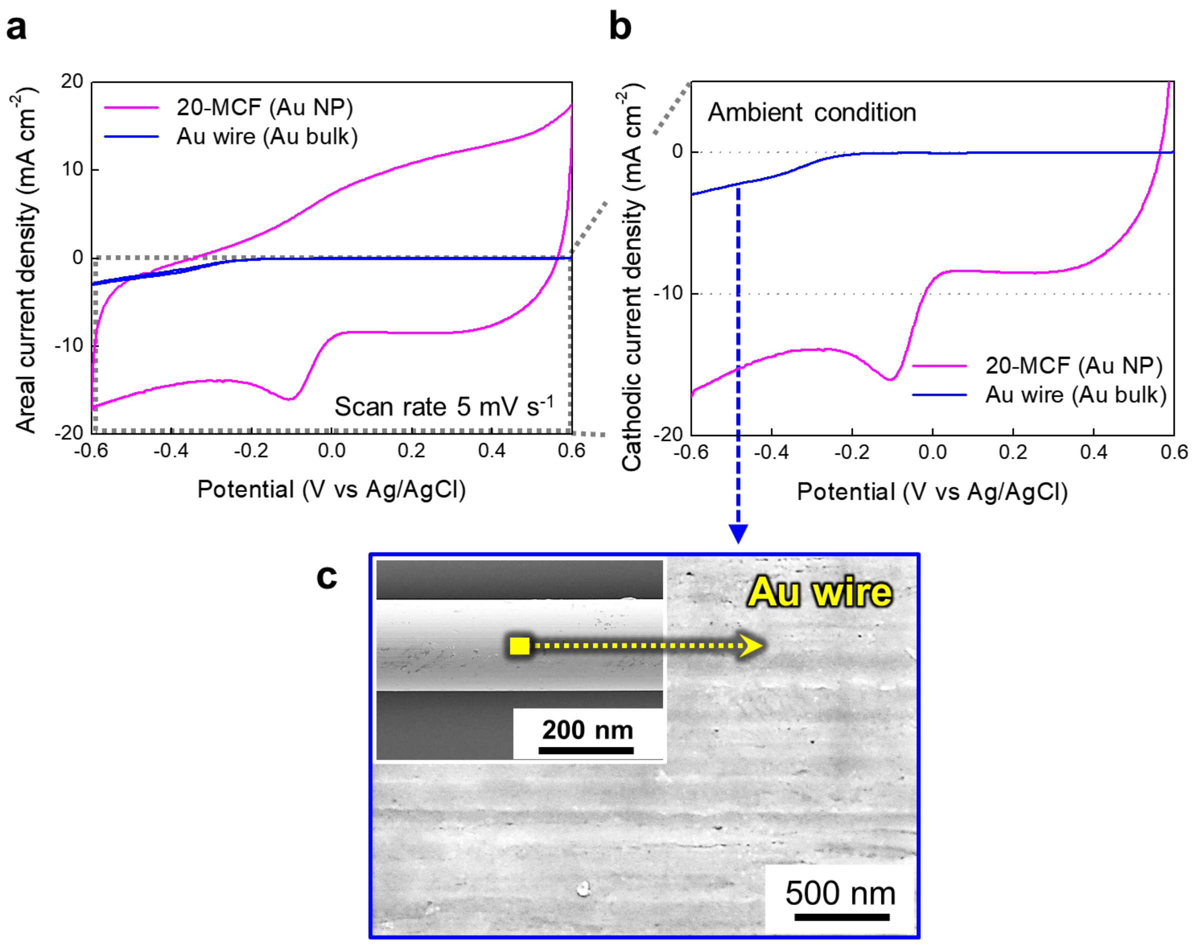

Supplementary Figure 8. Electrochemical performance of the metallic cotton fiber and Au wires. a, Cyclic voltammograms and **b**, cathodic areal current density curves of 20-MCF and nonporous Au wires with a diameter of 200 µm. **c**, Field emission-scanning electron microscopy (FE-SEM) images of nonporous Au wires (scale bar, 500 nm and 200 nm (inset)).

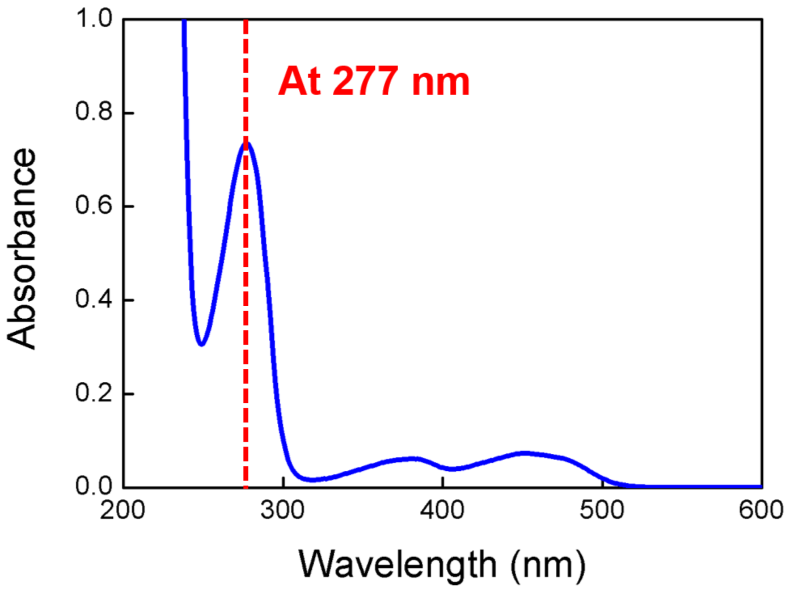

Supplementary Figure 9. UV-vis spectroscopy. UV-vis spectrum of GOx solution with a prominent absorption peak at 277 nm associated with the oxidized flavin cofactor in GOx[3].

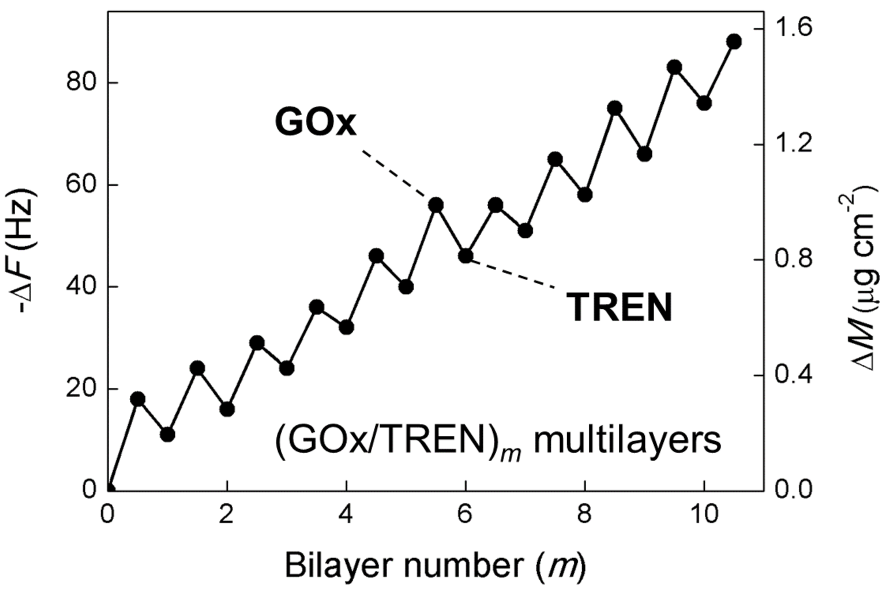

Supplementary Figure 10. Quartz crystal microbalance analysis. Frequency and mass changes of (anionic GOx/cationic TREN)$_m$ multilayers as a function of the number of bilayers (m) using QCM analysis. The concentrations of GOx and TREN in the aqueous PBS solution were adjusted to 5 and 1 mg ml^{-1}, respectively. Here, ΔF and ΔM indicate the frequency change and the mass change of the QCM electrode, respectively.

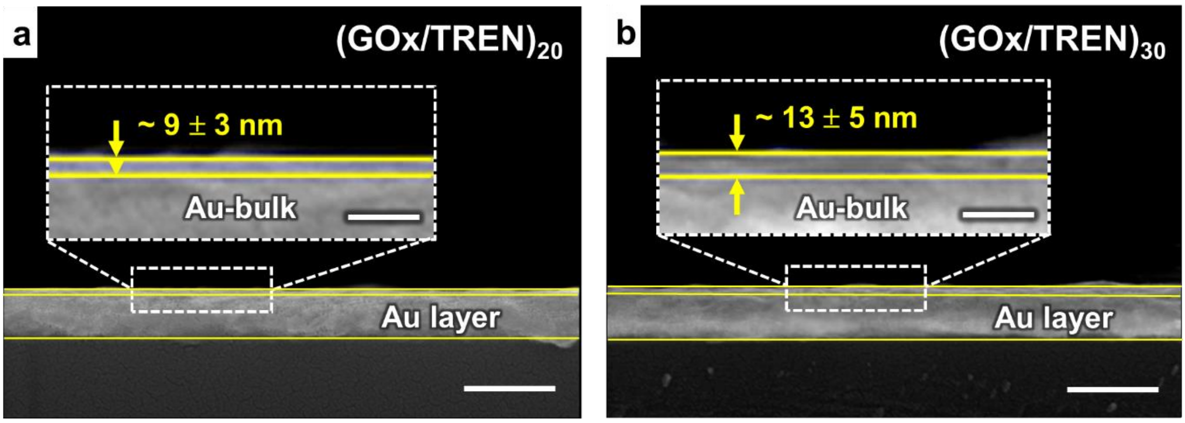

Supplementary Figure 11. Cross-sectional FE-SEM images for film thickness of **a,** $(GOx/TREN)_{20}$ and **b,** $(GOx/TREN)_{30}$ multilayers deposited onto the Au-coated Si wafer (scale bar, 200 nm and 50 nm (inset)).

.

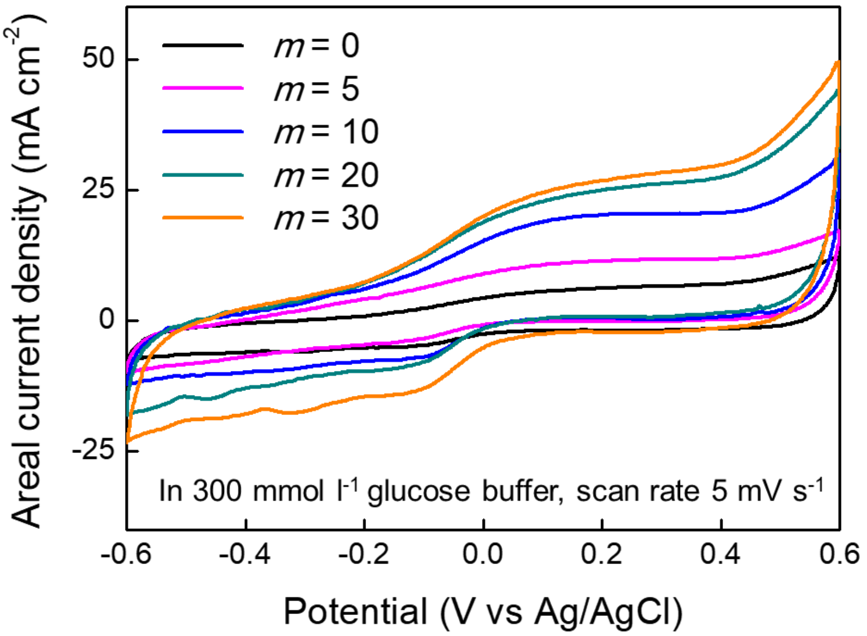

Supplementary Figure 12. CVs of the anode. Cyclic voltammograms of the *m*-GOx/20-MCF anode with increasing number of (GOx/TREN) bilayers (*m*). All the measurements were performed at a scan rate of 5 mV s^{-1} in PBS under ambient conditions.

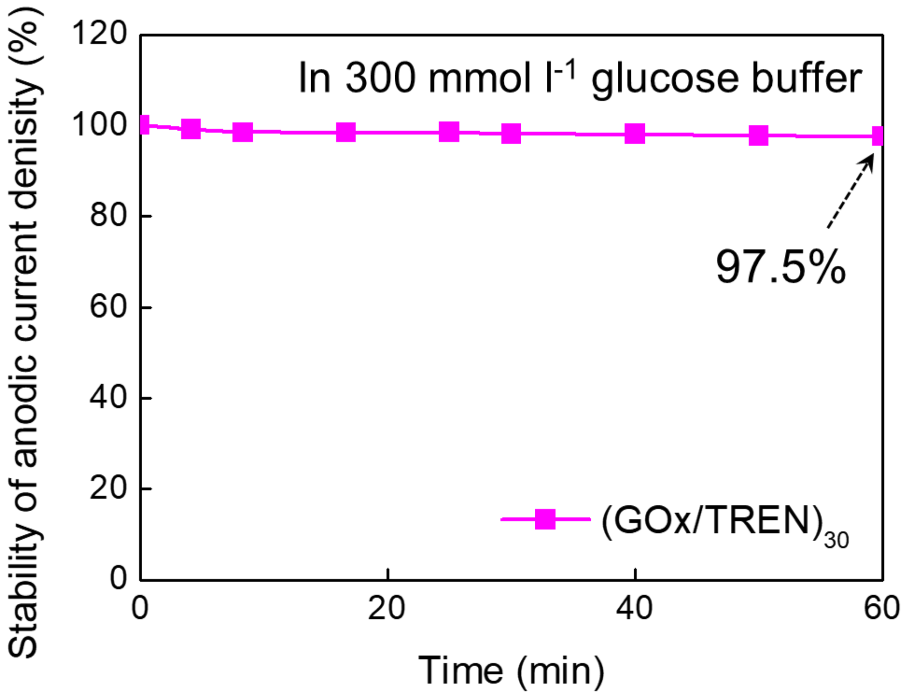

Supplementary Figure 13. Stability of anodic current density (%) for $(GOx/TREN)_{30}$/20-MCF anode electrode.

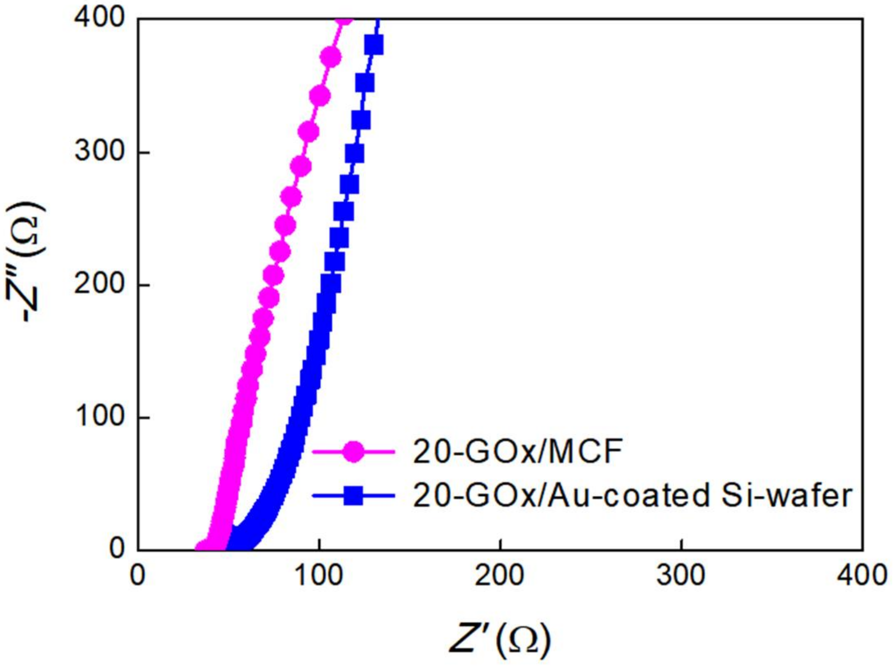

Supplementary Figure 14. Nyquist plots of the $(GOx/TREN)_{20}$ multilayers coated onto MCF (red solid circle) and nonporous plate substrate (i.e., Au-coated Si wafer substrate, blue solid square). In this case, ESR values of each electrode were measured to be ~38.6 Ω for 20-GOx/MCF and ~62.6 Ω for 20-GOx/Au-coated Si wafer electrodes, respectively.

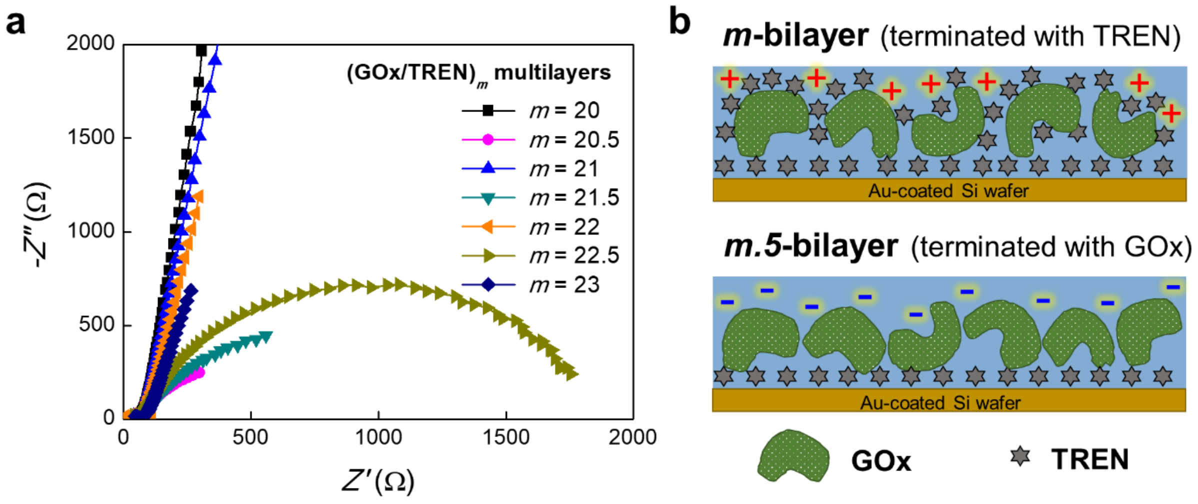

c

n	Terminated with	ESR (Ω)	R_{et}[a)] (Ω)
20	TREN	62.5	63
20.5	GOx	62.8	757
21	TREN	63.8	64
21.5	GOx	67.1	1415
22	TREN	66.1	66
22.5	GOx	66.8	1836
23	TREN	74.9	75

[a)]The interfacial electron transfer resistance between the charged interface of the electrode and the electrolyte.

Supplementary Figure 15. Outermost layer-dependent interfacial electron transfer kinetics (tested on the Au-coated Si wafer substrate). **a,** Nyquist plots of (GOx/TREN)$_m$ multilayers as a function of bilayer number (*m*). **b,** Representative schematics of (GOx/TREN)$_m$ multilayers having different surface charge as a function of outermost layer. **c,** Specific data sheet of (GOx/TREN)$_m$ multilayers.

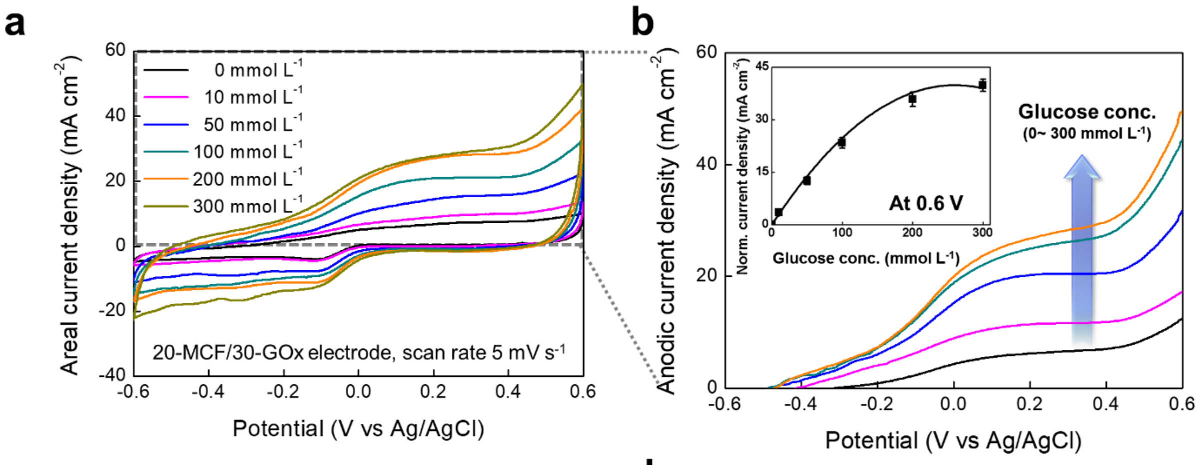

c

Glucose conc. (mmol L-1)	Measured current density level (mA cm-2)	Normalized current density level (mA cm-2)
0	10.2	0
10	13.6	3.4
50	22.9	12.7
100	33.2	23.0
200	42.7	32.5
300	50.1	39.9

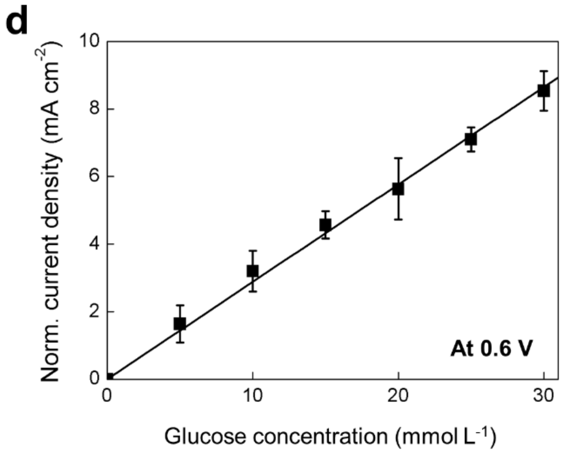

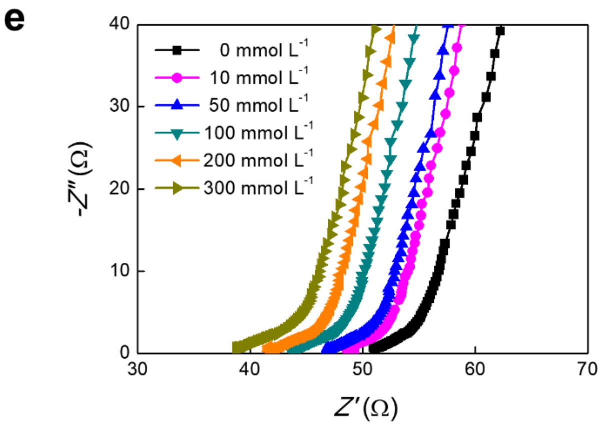

Supplementary Figure 16. Electrochemical performance of the anode. **a**, Cyclic voltammograms and **b**, anodic current density curves of the (30-GOx/20-MCF) anode as a function of glucose concentration. Inset: Normalized anodic current density levels of the (30-GOx/20-MCF) anode measured at +0.6 V as a function of glucose concentration. **c**, The normalized areal anodic current densities at +0.6 V. For example, the normalized current density of 39.9 mA cm^{-2} in a 300 mmol L^{-1} glucose solution was obtained by subtracting the current density of 10.2 mA cm^{-2} at +0.6 V in glucose-free phosphate-buffered saline (PBS)

from the measured current density level (50.1 mA cm^{-2}) at the same potential in a 300 mmol L^{-1} glucose. **d**, The plot of the normalized anodic current density levels at low glucose concentration (0 – 30 mmol L^{-1}). **e**, Nyquist plots of the (30-GOx/20-MCF) anode as a function of glucose concentration. The equivalent series resistance (ESR) values of the 30-GOx/20-MCF anode measured at 1 kHz decreased from 52 to 40 Ω if the glucose concentration was increased from 0 to 300 mmol L^{-1}. All the measurements were performed at a scan rate of 5 mV s^{-1} in a PBS under ambient conditions.

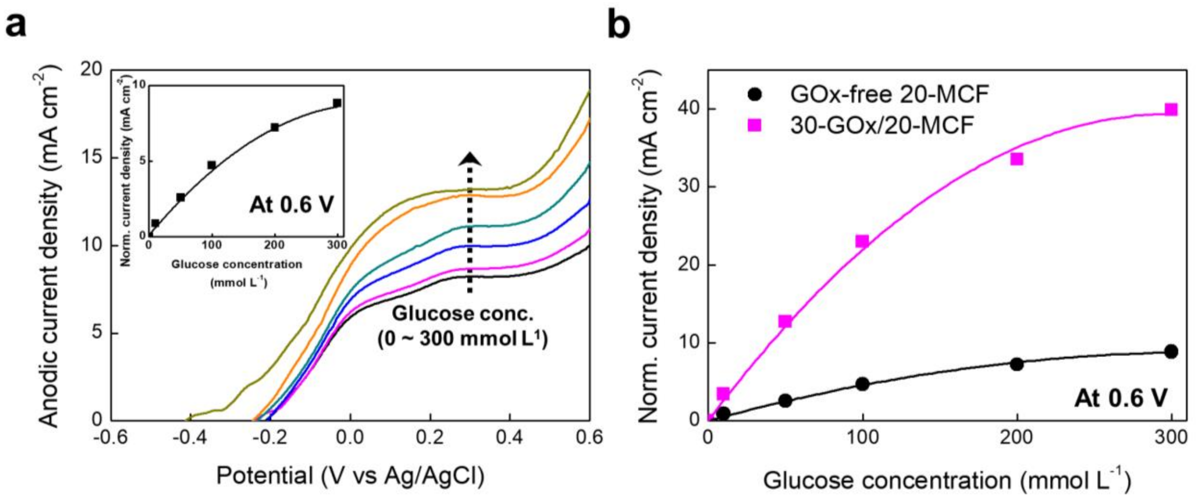

Supplementary Figure 17. Electrooxidation effect of bare MCF (i.e., GOx-free 20-MCF) for GOx. a, Anodic current density of GOx-free 20-MCF at the different glucose concentration of 10, 50, 100, 200, and 300 mmol L^{-1}, respectively. Inset indicates the normalized current density (in mA cm^{-2}) as a function of glucose concentration recorded at a potential of +0.6 V. **b**, Comparison of electrooxidation activities of GOx-free 20-MCF and 30-GOx/20-MCF anode electrodes at different glucose concentration. The electrochemical test was performed at a scan rate of 5 mV s^{-1} in a PBS (pH 7.4) under ambient condition. The normalized current density is obtained by subtracting the current density at +0.6 V measured in PBS without glucose. In this case, the oxidation of Au NPs loaded on the 20-MCF (~22%) was observed for GOx-free anode electrode.

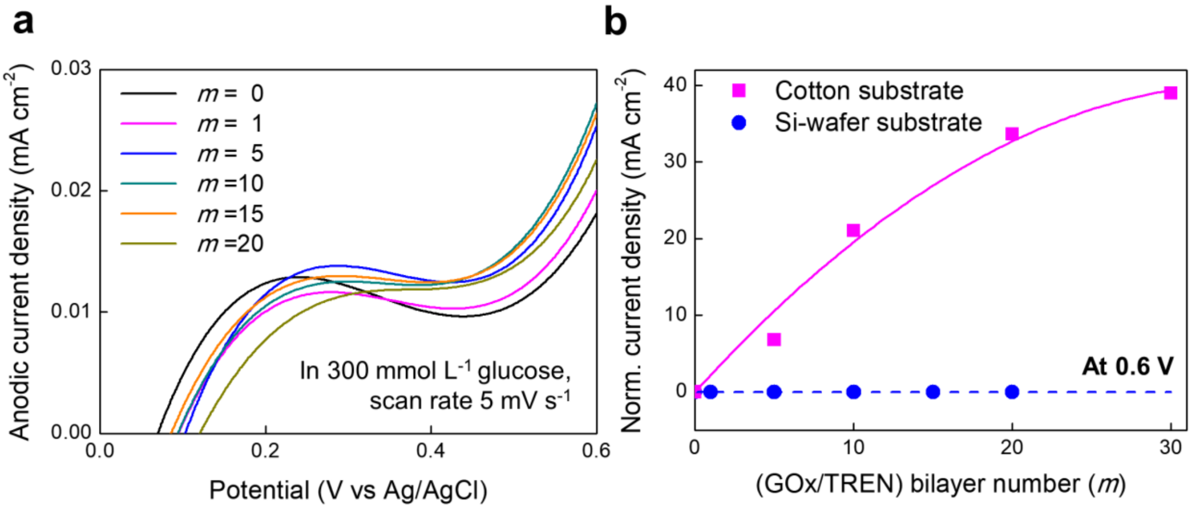

Supplementary Figure 18. Electrochemical performance of flat substrate-based anodes. **a**, Anodic current density curves. **b**, The normalized current density levels of cotton substrate- and Si wafer-based anodes measured at +0.6 V as a function of *m*. All the measurements were performed at a scan rate of 5 mV s^{-1} in a PBS containing 300 mmol L^{-1} glucose under ambient conditions. Here, the normalized anodic current density performance is ~0.01 mA cm^{-2} for the Si wafer substrate at 300 $mmol^{-1}$ glucose, compared to the value of the cotton substrate, ~33.6 mA cm^{-2} ($m = 20$).

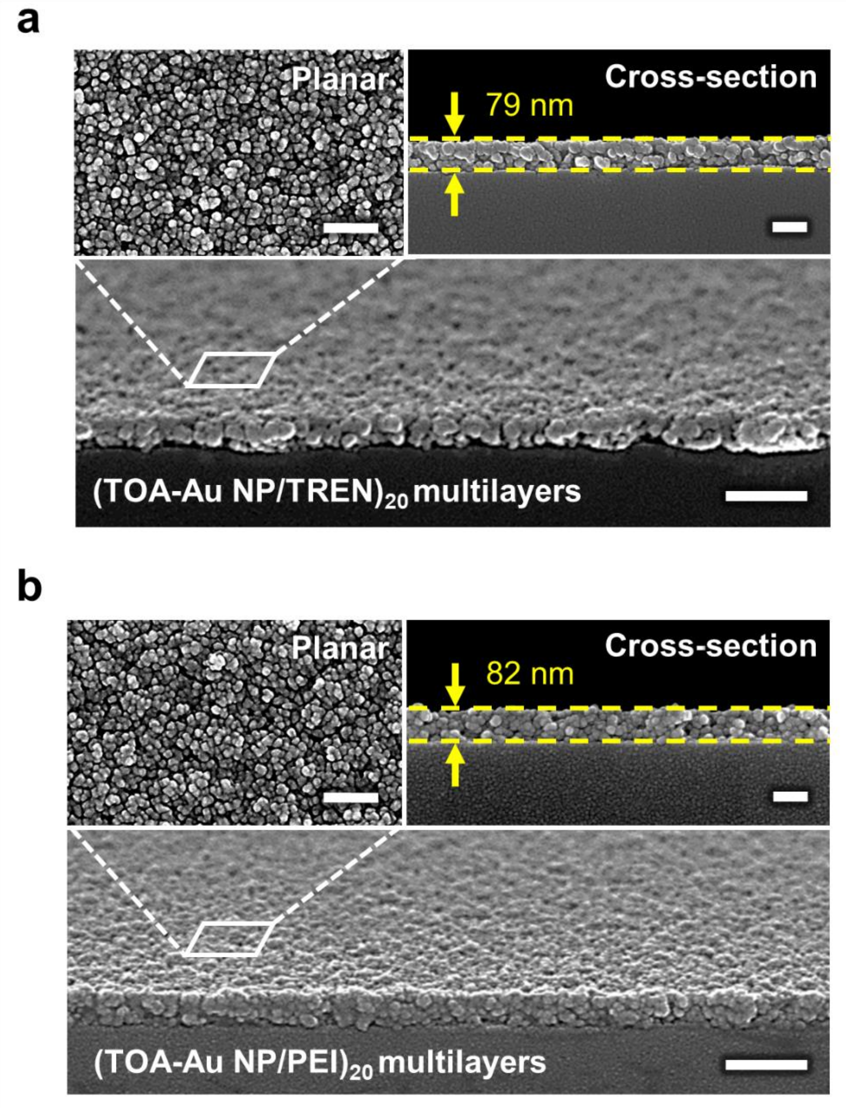

Supplementary Figure 19. Surface morphology comparison. The tilted SEM images of **a**, (TOA-Au NP/TREN)$_{20}$ (scale bar, 200 nm and 100 nm (insets)) and **b**, (TOA-Au NP/PEI)$_{20}$ multilayer-coated Si wafer. The insets of (a) and (b) show the planar (left side) and cross-section (right side) images (Scale bar, 200 nm and 100 nm (insets)).

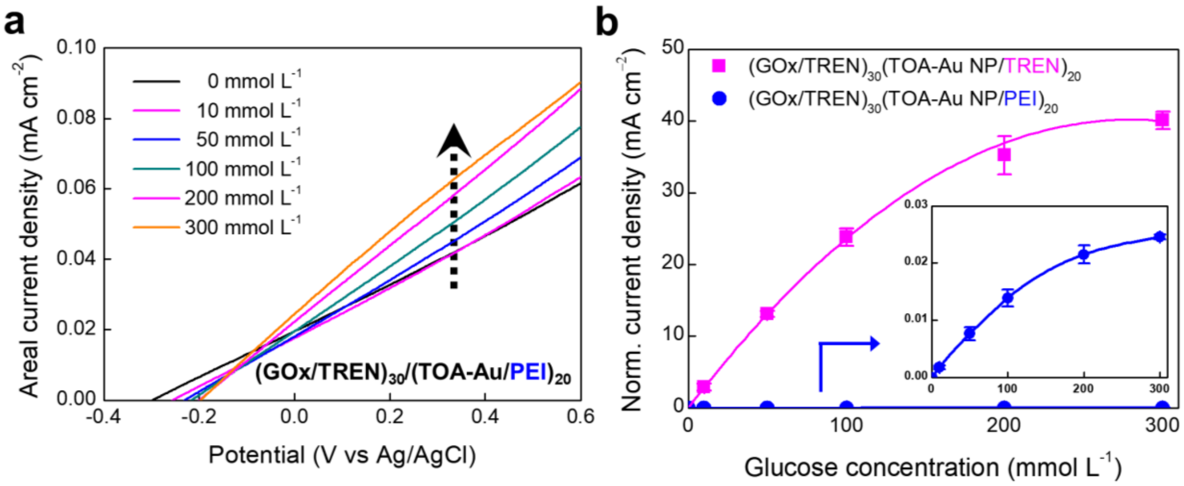

Supplementary Figure 20. Electrooxidation performance of (TOA-Au NP/PEI)$_{20}$-based MCF anode electrode. **a**, Anodic current density of (GOx/TREN)$_{30}$(TOA-Au NP/PEI)$_{20}$ electrode as a function of glucose concentration from 0 to 300 mmol L^{-1}. **b**, Comparison of the normalized current densities of (GOx/TREN)$_{30}$(TOA-Au NP/PEI)$_{20}$ and (GOx/TREN)$_{30}$(TOA-Au NP/PEI)$_{20}$ anode electrodes with different glucose concentration recorded at +0.6 V. Electrochemical test was conducted at a scan rate of 5 mV s^{-1} in a PBS (pH 7.4) under ambient condition. Here, the normalized anodic current density performance based on the highly-conductive (GOx/TREN)$_{30}$/(TOA-Au NP/TREN)$_{20}$ is ~1,600 folds higher than that of the less-conductive (GOx/TREN)$_{30}$/(TOA-Au NP/PEI)$_{20}$ electrode at 300 mmol L^{-1} glucose.

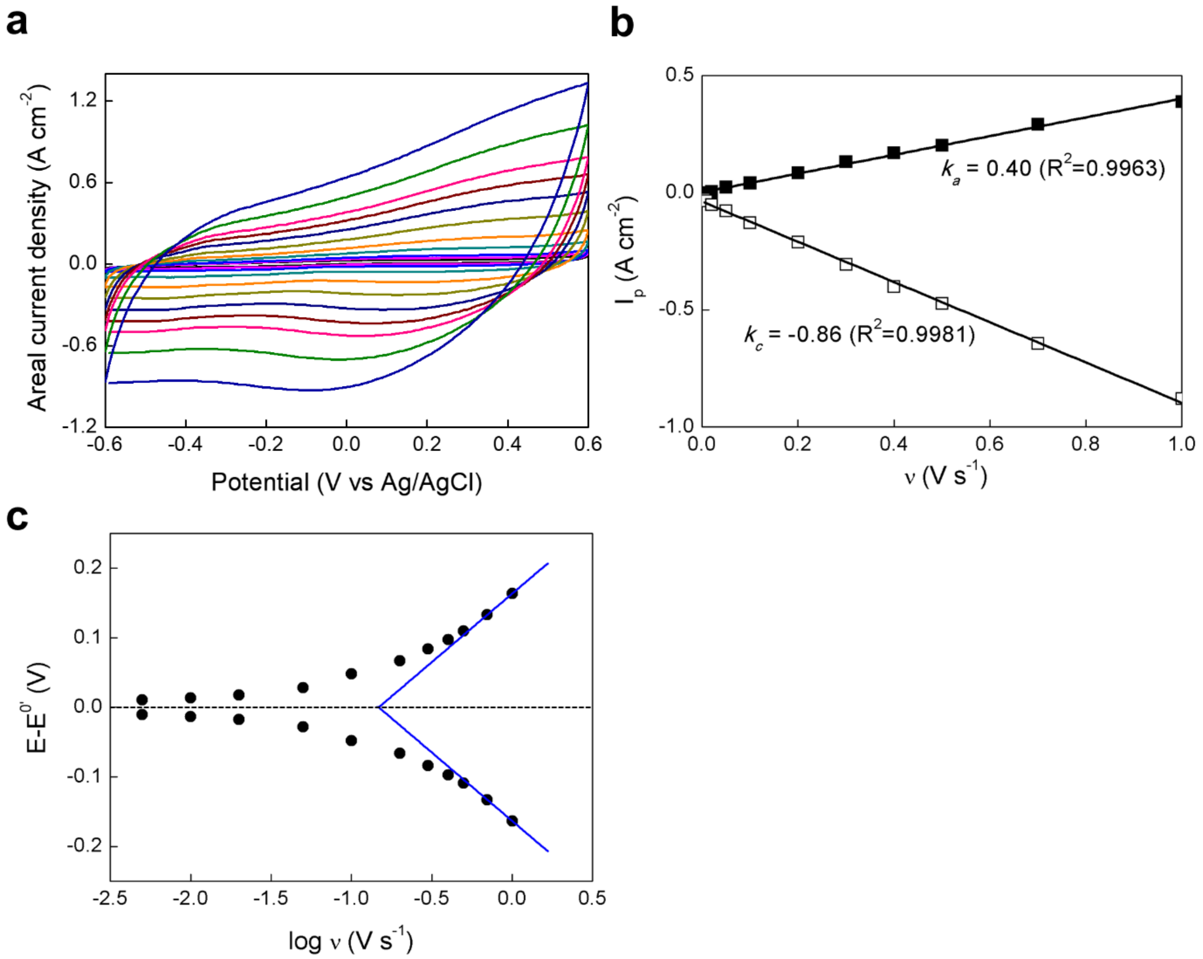

Supplementary Figure 21. Electrochemical response of the GOx-immobilized MCF anode electrode. a, Scan rate (v)-dependent CVs of the anodic electrode, 30-GOx/20-MCF, in 300 mmol L^{-1} glucose buffer condition. Scan rates (from inner to outer of CV curves) were defined as 0.005, 0.01, 0.02, 0.05, 0.1, 0.2, 0.3, 0.4, 0.5, 0.7, and 1.0 V s^{-1}. **b,** The plot of peak currents of 30-GOx/20-MCF with increasing the scan rate from 0.005 to 1 V s^{-1}. **c,** The plot of potential ($E-E^{0}$') versus log (v).

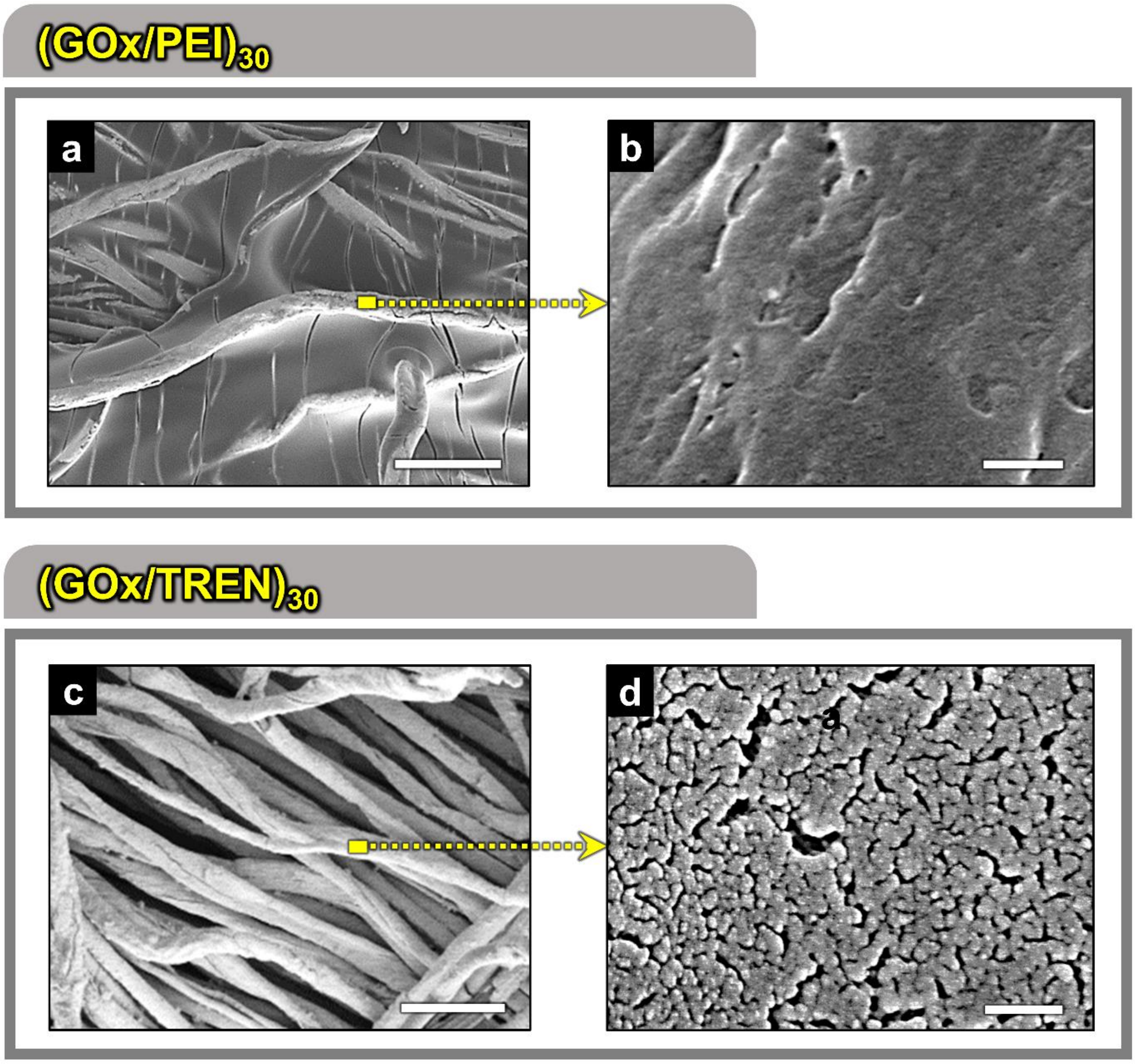

Supplementary Figure 22. Surface morphologies of the anode. FE-SEM images of the (30-GOx/20-MCF) anodes incorporating **a**, **b** (scale bar, 100 μm and 200 nm, respectively), PEI [$(GOx/PEI)_{30}$ on 20-MCF] and **c**, **d**, TREN linkers [$(GOx/TREN)_{30}$ on 20-MCF] (scale bar, 100 μm and 200 nm, respectively).

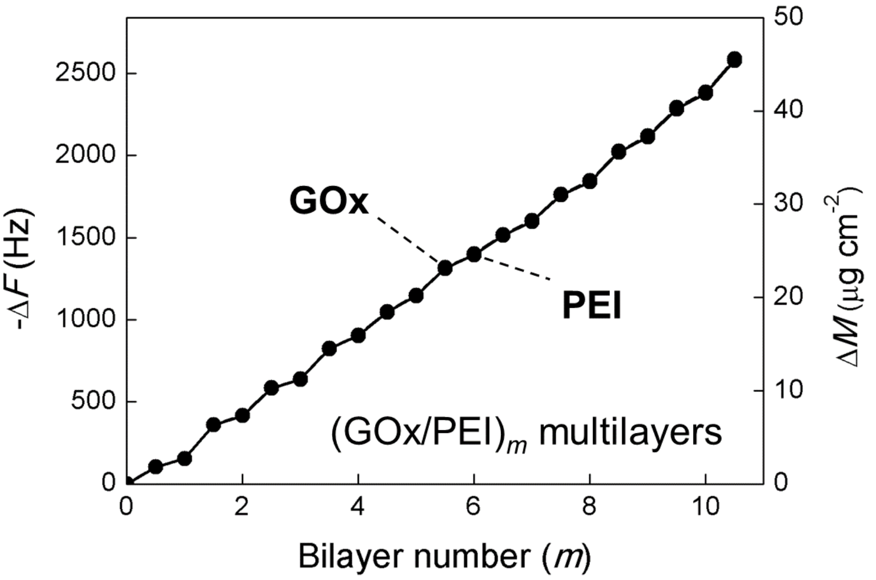

Supplementary Figure 23. Quartz crystal microbalance analysis of the GOx/PEI multilayers. Frequency (ΔF) and mass changes (ΔM) of $(GOx/PEI)_m$ as a function of the number of bilayers (m).

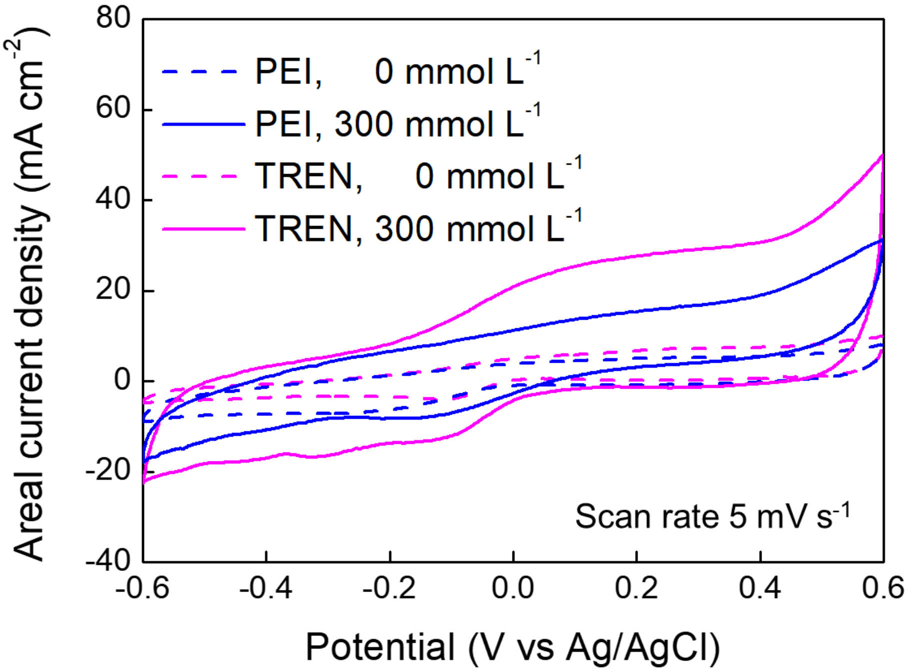

Supplementary Figure 24. CVs of linker type-dependent anodes. CV curves of $(GOx/PEI)_{30}$ and $(GOx/TREN)_{30}$ on 20-MCF in the absence or presence of glucose (300 mmol L^{-1}) in PBS under ambient conditions.

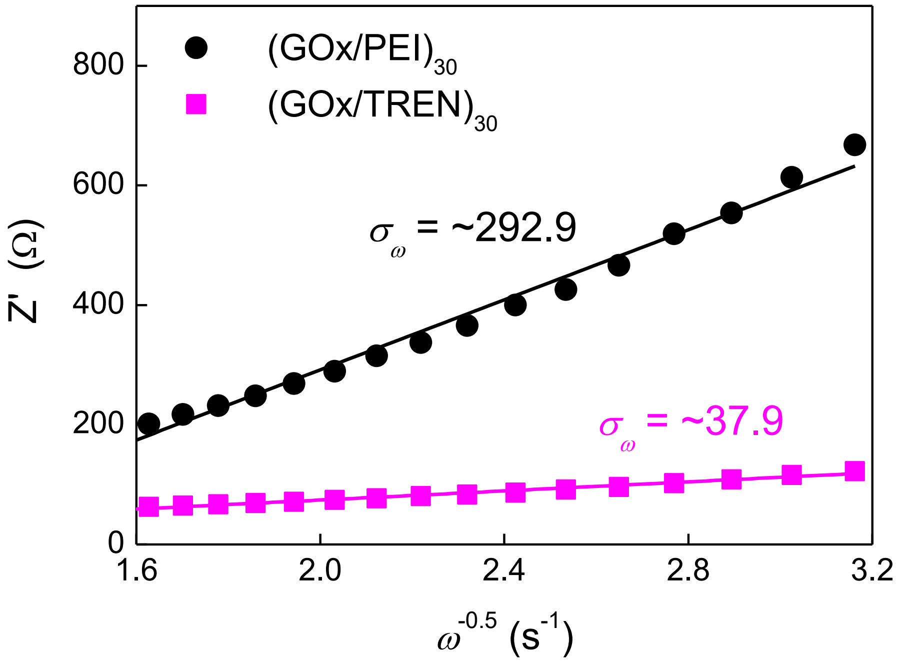

Supplementary Figure 25. Warburg impedance coefficient plot. Plots of $Z'(\Omega)$ versus $\omega^{-0.5}$ ($s^{-0.5}$) for (GOx/PEI)$_{30}$ and (GOx/TREN)$_{30}$ on 20-MCF.

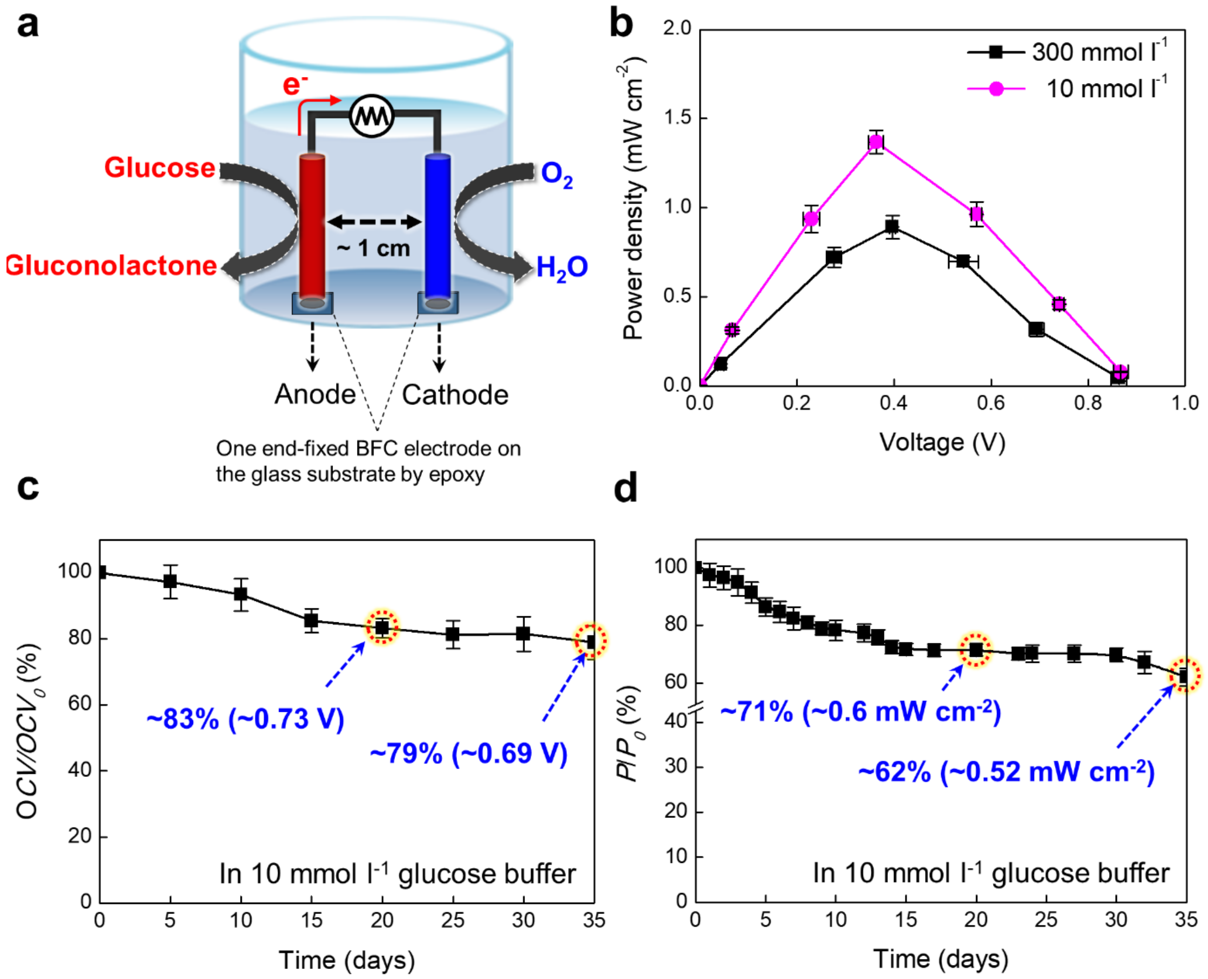

Supplementary Figure 26. Performance of the metallic cotton fiber-biofuel cells. a, Schematic diagram showing the complete membraneless hybrid MCF-BFC. One end of each BFC electrode was fixed by epoxy on the glass substrate for avoiding electrical shortage during the continuous operation. **b**, Power output of the complete BFC with an external resistor (1 kΩ ~ 10 MΩ) consisting of the 30-GOx/20-MCF anode and 20-MCF cathode as a function of voltage. **c**, Relative voltage retention (OCV/OCV_0) of the complete MCF-BFC, which is measured under open-circuit system. **d**, Relative power retention (P/P_0) of the complete MCF-BFC in 10 mmol l^{-1} glucose buffer during 35 days. In this case, the power output changes of MCF-BFCs with external resistors were continuously measured as a function of time. The error bars show the standard deviation from the mean value of power densities for three to five independent experiments (**b**, **c**, **d**).

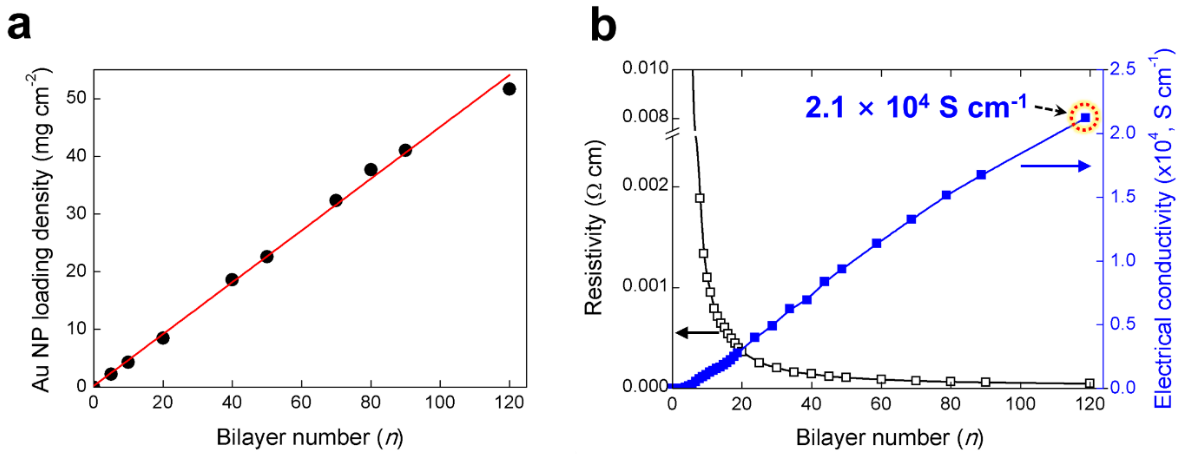

Supplementary Figure 27. Physical and electrical properties of *n*-MCFs. a, TOA-Au NP/TREN loading density and **b**, resistivity and electrical conductivity of *n*-MCFs as a function of the number of bilayers (n), up to n = 120. The mass percentage of the Au NPs within the TOA-Au NP/TREN multilayer was measured to be ~98%. This was obtained through heat treatment (at 150 °C in air) of the TOA-Au NP/TREN multilayer-deposited QCM electrode to eliminate the organic components.

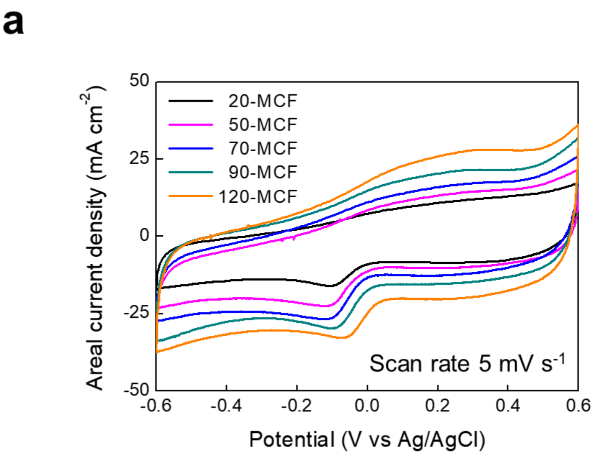

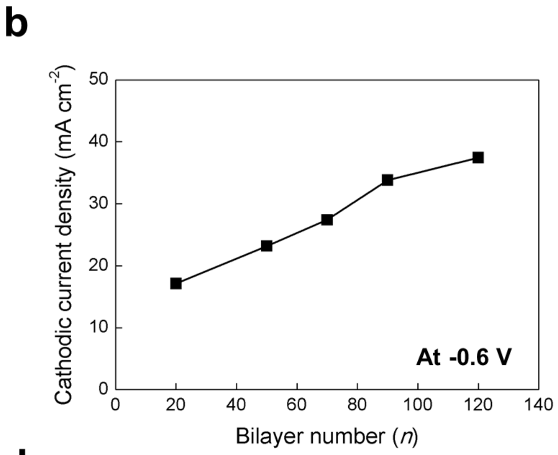

c

Cathode	Measured current density level (mA cm^{-2})	Current density level under N_2 condition (mA cm^{-2})	Normalized current density level (mA cm^{-2})
20-MCF	17.1	7.2	9.9
50-MCF	23.2	12.3	10.9
70-MCF	27.4	15.4	12.0
90-MCF	33.8	18.3	15.5
120-MCF	37.5	19.9	17.6

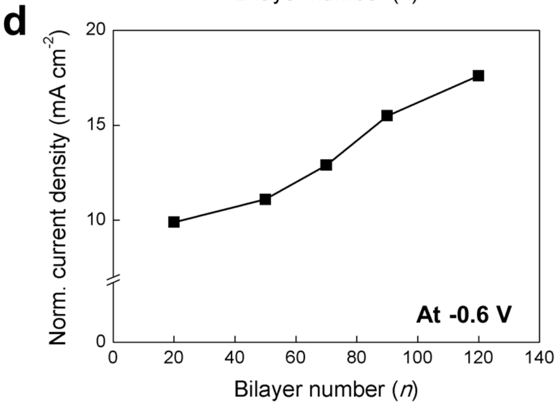

Supplementary Figure 28. CVs of the metallic cotton fiber cathodes. a, CV curves of the *n*-MCF cathodes with increasing number of bilayers (*n*) from 20 to 120. **b**, The measured areal cathodic current density levels of the *n*-MCF cathodes measured at −0.6 V. **c**, **d**, The normalized current density levels at −0.6 V from 20-MCF to 120-MCF. All the measurements were performed at a scan rate of 5 mV s^{-1} in a PBS under ambient conditions.

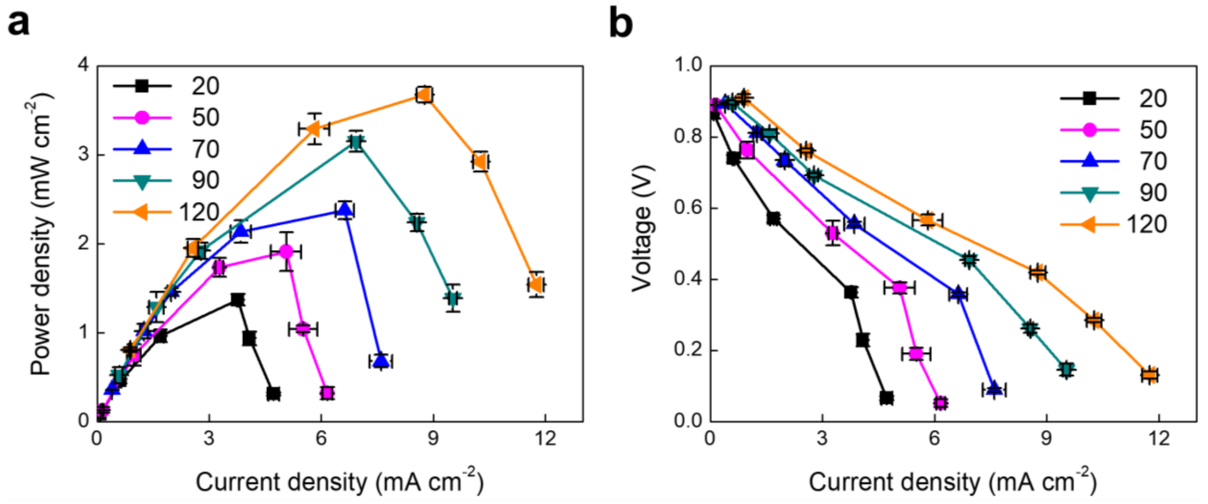

Supplementary Figure 29. Characteristics of the MCF-BFCs by measuring the current output through external resistors (in the range of 1 kΩ ~ 10 MΩ) to control cell potential. **a**, The *power (P) – current (I)* profiles of the hybrid BFC. **b**, The *V–I* profiles of the *n*-MCF hybrid BFC as a function of *n*. All the measurements were performed in PBS containing glucose (300 mmol L^{-1}) under ambient conditions at 37 °C. All error bars show the standard deviation from the mean value of power densities for three to five independent experiments.

Supplementary Table 1. Power performance and stability of various BFCs. Power density and operational stability of various BFCs reported to date.

Host electrode	Catalysts[b)] Anode/Cathode	Fuel Anode/Cathode	Operation condition	P_{max} (mW cm^{-2})	OCV (V)	Ref.
**MCF[a)]**	**GOx/Au NP**	**Glucose/O_2**	**PBS (pH 7.4) at 37º C, ambient**	**3.7**	**~0.9**	**Our work**
CNT fiber	GOx/BOD	Glucose/O_2	PBS (pH 7.0) at 37 ºC, air	0.74	0.83	**4**
CNT yarn	GOx/BOD	Glucose/O_2	PBS (pH 7.2) at 37 ºC, O_2	2.18	0.7	**5**
CNT film	GOx/Pt_{bulk}[c)]	Glucose/O_2	PBS (pH 7.4), air	1.34	~0.8	**6**
Compressed CNTs	GOx-Cat/Lac	Glucose/O_2	PBS (pH 7.0)	1.25	0.95	**7**
3D Au NP (on carbon paper)	FDH/BOD	Fructose/O_2	0.1 M acetate buffer (pH 6.0) at 25 ºC, O_2	0.87	~0.7	**8**
SWNT (on glassy carbon)	GDH/Lac	Glucose/O_2	PBS (pH 6) at 20 ºC, ambient	0.0095	0.8	**9**
Graphene/Au NP Hybrid (on Au substrate)	FDH/Lac	Formic acid/O_2	PBS (pH 6.0)	1.96	0.95	**10**
Nafion/poly(vinyl pyrolidone) compound nanowire (on Au electrode)	GOx/Lac	Glucose/O_2	PBS (pH 7.0)	0.03	0.23	**11**
Catecholamine polymers (on Au disk)	GOx/Carbon rod	Glucose/O_2	PBS (pH 7.0)/ KMnO4 + H_2SO_4	1.62	1.09	**12**
Au NP/PANI (polyaniline) network (on glassy carbon)	GOx/Lac	Glucose/O_2	PBS (pH 7.4) at 20 ºC	0.685	0.76	**13**
Enzyme cluster composite (on carbon paper)	GOx/Pt_{bulk}[c)]	Glucose/O_2	1.0 M PBS (pH 7.4)	1.62	~0.8	**14**
Compressed MWNT[d)] /enzyme composite	GOx/Lac	Glucose/O_2	In the abdominal cavity of a rat	0.193	0.57	**15**
Graphene and SWNT[d)] cogel	GOx/BOD	Glucose/O_2	Air-saturated sodium phosphate buffer (0.1 M, pH 7.0) with 100 mM glucose	0.19	0.61	**16**

CS/CNC film (on glassy carbon)	GOx/Pt sheet	Glucose/O_2	Air-saturated PBS (pH 7.2) containing 10 mM glucose	0.06	0.59	**17**
Carbon fiber sheets with membrane film using ANQ[e)]	GDH/BOD	Glucose/O_2	Sodium phosphate buffer (1 M, pH 7)	3.0	0.5	**18**
(GOx or Lac)/PEI/CNT composite consisting of GA[f)]	GOx/Lac	Glucose/O_2	PBS (pH 3), O_2	0.102	~0.43	**19**

[a)] MCF: metallic cotton fiber

[b)] The abbreviations for different enzymes: glucose oxidase (GOx), laccase (Lac), bilirubin oxidase (BOD), fructose dehydrogenase (FDH), glucose dehydrogenase (GDH), and catalase (Cat).

[c)] Pt_{bulk}: Commercial Pt bulk electrode

[d)] MWNT: multi-walled carbon nanotube, SWNT: single-walled carbon nanotube, CS/CNC: chitosan/carbon nanochips

[e)] 2-amino-1,4-naphthoquinone (ANQ) as an electron mediator

[f)] PEI: poly(ethyleneimine), GA: glutaraldehyde

Supplementary References

1. Lopes, J. H., Ye, S., Gostick, J. T., Barralet, J. E. & Merle, G. Electrocatalytic oxygen reduction performance of silver nanoparticle decorated electrochemical exfoliated graphene. *Langmuir* **31,** 9718–9727 (2015).

2. Zhao, S. et al. Three-dimensional graphene/Pt nanoparticle composites as freestanding anode for enhancing performance of microbial fuel cells. *Sci. Adv*. **1,** 1–8. (2015).

3. Zhou, K., Zhu, Y., Yang, X. & Li, C. Electrocatalytic oxidation of glucose by the glucose oxidase immobilized in graphene-Au-nafion biocomposite. *Electroanalysis* **22,** 259–264 (2010).

4. Gao, F., Viry, L., Maugey, M., Poulin, P. & Mano, N. Engineering hybrid nanotube wires for high-power biofuel cells. *Nat. Commun.* **1,** 2 (2010).

5. Kwon, C. H. et al. High-power biofuel cell textiles from woven biscrolled carbon nanotube yarns. *Nat. Commun.* **5,** 3928 (2014).

6. Hyun, K. H., Han, S. W., Koh, W. –G & Kwon, Y. Fabrication of biofuel cell containing enzyme catalyst immobilized by layer-by-layer method. *J. Power Sources* **286,** 197–203 (2015).

7. Zebda, A. et al. Mediatorless high-power glucose biofuel cells based on compressed carbon nanotube-enzyme electrodes. *Nat. Commun.* **2,** 370 (2011).

8. Murata, K., Kajiya, K., Nakamura, N. & Ohno, H. Direct electrochemistry of bilirubin oxidase on three-dimensional gold nanoparticle electrodes and its application in a biofuel cell. *Energy Environ. Sci.* **2,** 1280–1285 (2009).

9. Yan, Y., Zheng, W., Su & Mao, L. Carbon-nanotube-based glucose/O_2 biofuel cells. *Adv. Mater.* **18,** 2639–2643 (2006).

10. Gai, P. et al. A nitrogen-doped graphene/gold nanoparticle/formate dehydrogenase bioanode for high power output membrane-less formic acid/O_2 biofuel cells. *Analyst*

140, 1822–1826 (2015).

11. Pan, C. et al. Generating electricity from biofluid with a nanowire-based biofuel cell for self-powered nanodevices. *Adv. Mater.* **22,** 5388–5392 (2010).

12. Chen, C. et al. High-performance amperometric biosensors and biofuel cell based on chitosan-strengthened cast thin films of chemically synthesized catecholamine polymers with glucose oxidase effectively entrapped. *Biosens. Bioelectron.* **26,** 2311–2316 (2011).

13. Mishra, P. et al. Electrocatalytic biofuel cell based on highly efficient metal-polymer nanoarchitectured bioelectrodes. *Nano Energy* **39,** 601–607 (2017).

14. Chung, Y., Christwardana, M., Tannia, D. C., Kim, K. J. & Kwon, Y. Biocatalyst including porous enzyme cluster composite immobilized by two-step crosslinking and its utilization as enzymatic biofuel cell. *J. Power Sources* **360,** 172–179 (2017).

15. Zebda, A. et al. Single glucose biofuel cells implanted in rats power electronic devices. *Sci. Rep.* **3,** 1516 (2013).

16. Campbell, A. S. et al. Membrane/Mediator-free rechargeable enzymatic biofuel cell utilizing graphene/single-wall carbon nanotube cogel electrodes. *ACS Appl. Mater. Interfaces* **7,** 4056−4065 (2015).

17. Kang, Z. et al. Direct electrochemistry and bioelectrocatalysis of glucose oxidase in CS/CNC film and its application in glucose biosensing and biofuel cells. *RSC Adv.* **7,** 4572–4579 (2017).

18. Sakai, H. et al. A high-power glucose/oxygen biofuel cell operating under quiescent condition. *ECS Trans.* **16,** 9–15 (2009).

19. Christwardana, M., Kim, K. J. & Kwon, Y. Fabrication of Mediatorless/Membraneless glucose/oxygen based biofuel cell using biocatalysts including glucose oxidase and laccase enzymes. *Sci. Rep.*, **6,** 30128 (2016).
